# Food and plastic waste generation at a large-scale religious festival and implications for sustainable management

**DOI:** 10.1177/0734242X251385955

**Published:** 2025-11-03

**Authors:** Hana Kadum, Hussien L. Algboory, Nameer Khairullah Mohammed, Belal J. Muhialdin, Viachaslau Filimonau

**Affiliations:** 1 Biology Department, College of Science, Al-Muthana University, Samawah, Iraq; 2 University of Information Technology and Communications Biomedical Information College, Baghdad, Iraq; 3 Department of Food Science, Faculty of Agriculture, Tikrit University, Tikrit, Iraq; 4 Nutrition and Food Science Department, California State Polytechnic University, Pomona, CA, USA; 5 Business School, University of Surrey, Guildford, UK

**Keywords:** Mega event, religious tourism, pilgrimage, solid waste generation, hospitality, sustainability

## Abstract

Large-scale religious events generate considerable amounts of solid waste calling for dedicated research to quantify wastage, explore its drivers, understand stakeholder perceptions and design effective management strategies. Responding to this call, this study investigated the generation of food and plastic waste during the 2023 Arba’een pilgrimage in Karbala, Iraq, one of the world’s largest annual religious festivals. A mixed-methods research design was employed combining quantitative waste audits, conducted over 20 days at selected mawkibs (volunteer-run foodservice stations) and municipal waste disposal points, with qualitative semi-structured interviews (*n* = 60) involving mawkib owners, pilgrims, religious leaders and municipal authorities. Audits revealed substantial waste: 7900 tonnes of food and 4000 tonnes of plastic. Per pilgrim, 0.36 kg of food and 0.18 kg of plastic were generated, accounting for 0.72% and 0.58% of Iraq’s total annual hospitality food waste and all-sectors-total plastic waste, respectively. Interviews explored such thematic areas as waste drivers, behavioural practices and responsibility attribution. Findings highlighted a ‘blame game’ dynamic between mawkibs and pilgrims regarding wastage. Religious leaders cited a moral tension, noting how observed excess in consumption contrasted sharply with Islamic values of modesty and resource conservation, while authorities stressed logistical constraints. This study provides novel empirical data, highlighting the complex interplay between traditional Islamic hospitality and sustainability. It outlines scope for waste reduction interventions, such as portion control at mawkibs and promoting alternative serving materials, such as bio-plastics, for future large-scale religious events.

## Introduction

The accelerating scale and frequency of large-scale events, ranging from religious pilgrimages to sporting competitions, present a critical challenge to sustainable waste management (Parkes et al., 2016). Such events, attracting many participants within limited timeframes and restricted localities, generate disproportionate amounts of solid waste, particularly food and plastics (Gaffney, 2013). For example, large-scale gatherings produce up to 7 kg of food and plastic waste per participant daily, which is significantly higher than wastage among residents (Zafari et al., 2025). Concurrently, managing waste at large-scale events is challenging not only due to their size and temporal nature but also because of logistical issues and underdeveloped infrastructure (Mariyam et al., 2024). This often results in significant downstream environmental degradation, such as accelerated landfill saturation, soil and waterway pollution from leachate, and the release of greenhouse gas (GHG) emissions from decomposing organic waste (Rafiee et al., 2018). Such negative consequences emphasise the urgent need for effective waste management interventions to safeguard both the environment and public health, especially in developing nations where environmental considerations are often overlooked at the stage of large-scale events’ planning and staging (Chakrabarty, 2020). To enhance environmental sustainability of large-scale events, it is necessary to understand the dynamics of waste generation alongside its drivers and, consequently, develop effective mitigation interventions (Martinho et al., 2018).

Among various large-scale events, religious pilgrimages/festivals present distinct waste management challenges (Abdulredha et al., 2020a). These events are grounded in cultural and religious practices striving to demonstrate exceptional, celebratory hospitality (Gonzalez et al., 2019). Such hospitality is often exemplified by communal feeding and ritualistic food offerings which can, due to their generosity, lead to food waste (Gupta and Basak, 2018). Besides, provision of food and food consumption behaviour at religious gatherings are frequently shaped by complex social norms (Fieldhouse, 1995), moral considerations (Minton et al., 2019) and identity signalling (Sosis, 2006) within the religious and wider social context. These factors influence food acceptance by religious event attendees, their food choice and leftover generation (Teng et al., 2023).

For instance, in an attempt to attract pilgrims, thus increasing brand recognition and reputation, hospitality providers can enlarge meals, thereby prompting leftovers (Filimonau et al., 2023). Besides, if an event is staged in a remote area, in a hot season or because of other logistical reasons, including convenience and hygiene, considerable generation of plastic waste (for instance, water bottles) can also occur (Kutralam-Muniasamy and Shruti, 2022). Researching waste generation and management at religious festivals is therefore crucial not only for mitigating growing environmental impacts of large-scale events but also for understanding the delicate interplay of religious practice and sustainability (Elgammal and Alhothali, 2021). The insights provided by such research can inform the design of more effective waste reduction strategies, while respecting the sanctity of religious festivals and promoting environmental responsibility among its participants and organisers (Rafiee et al., 2018).

The Arba’een pilgrimage in Iraq provides an interesting case to study the complexities of waste management at large-scale religious festivals. This annual pilgrimage commemorates the 40th day after the martyrdom of Imam Husayn ibn Ali, the grandson of the Prophet Muhammad and the third Shia Imam, which is a major event in Islamic history symbolising sacrifice, justice and resistance against oppression (Husein, 2018). Arba’een, usually attracting over 20 million visitors, represents a key annual pilgrimage in the world. It is over ten times larger than Hajj which recorded circa 1.84 million pilgrims in 2023 (Statista, 2024). Arba’een is characterised by immense acts of hospitality and communal sharing enabled by *mawkibs*, traditional Shiite service stations providing free food and beverages to pilgrims (Al-Ansari et al., 2021). Research demonstrates that this provision results in significant wastage of food and plastics (Filimonau et al., 2022b). Besides, this festival’s limited timeframe and restricted geographical location in the city of Karbala, where the pilgrimage ends, coupled with the region’s existing waste management infrastructure challenges, amplify the need for targeted solutions to mitigate the occurrence of food and plastic waste (Abdulredha et al., 2020b). An in-depth analysis of solid waste generation patterns during the Arba’een pilgrimage can offer insights aiding in understanding the complexity of solid waste management at other large-scale religious events globally.

Despite the growing recognition of the significant environmental impacts associated with staging large-scale events (Cavallin Toscani et al., 2022), empirical research on solid and, especially food and plastic, waste generation and management at major religious festivals remains limited. For example, a systematic literature review of research on pilgrimage and religious tourism published between 1979 and 2024, while acknowledging increasing scholarly interest in investigations related to sustainability, revealed no single empirical study focusing on solid waste management (Singh and Bhuyan, 2024). Likewise, Foltin et al. (2020) and Shinde and Olsen (2020) underlined the importance of accurate measurement of the mounting environmental externalities of religious and spiritual events, including wastage, but did not assess their magnitude. There is a distinct lack of empirical, in-situ studies that directly quantify food and plastic waste generation and explore the determinants of its occurrence and effective management during large-scale religious events, such as the Arba’een pilgrimage. Previous scholarly work on solid waste at religious events has predominantly relied on secondary data or self-reported figures, which inherently limits insights into real-time waste dynamics. Several notable exceptions featuring empirical investigations of solid waste generation and management at large-scale religious events are as follows.

El Hanandeh (2013) estimated the carbon footprint of Hajj, including emissions attributed to waste generation and management. However, their study used secondary data to derive the estimates rather than direct, on-site measurements. Campos et al. (2022) quantified the carbon footprint of the Camino Lebaniego pilgrimage (Spain) using secondary and self-reported data on consumption of food, paper, plastic and glass by accommodation providers and their guests. Hence, this study focused on the environmental implications of hospitality services provision during pilgrimage rather than on the event itself. Lastly, specifically in the context of the Arba’een pilgrimage, Abdulredha et al. (2017) collected samples of unprocessed solid waste generated during the 2016 event. Their study found that the samples were dominated with food, paper and plastic residues, thus revealing priority fractions for reduction. To complement these results, Abdulredha et al. (2018) used self-reported data from hotel owners/managers to estimate the amount of solid waste generated in accommodation facilities of Karbala during the 2016 pilgrimage. Their study established that, an ‘average’ pilgrim may have generated up to 0.89 kg of solid waste (mostly food, plastic and paper) per day of the event. Lastly, in a follow-up study, Abdulredha et al. (2020b) interviewed policy officers responsible for solid waste management in the city of Karbala to understand the dynamics of waste generation and disposal during the 2016 pilgrimage. Their findings showed that solid waste generated during the Arba’een event was mismanaged with only 70% of it collected for disposal via uncontrolled landfilling and with only 5% of it recycled by informal waste pickers. This study recognised the need for more nuanced investigation of the solid waste challenge during the Arba’een pilgrimage given significant detrimental impacts of its improper management on the environment and local communities in Karbala.

The gap in empirical research on food and plastic waste generation during major religious events is concerning given the unique socio-cultural dynamics and environmental implications associated with religious pilgrimages. The lack of research can be explained by several factors. Firstly, religious festivals are often deeply embedded in cultural and spiritual contexts, making them sensitive areas for academic inquiry (Shinde and Olsen, 2020). Academics may hesitate to commission studies that could be perceived as intrusive or disrespectful from the cultural/religious perspective. Secondly, the logistical complexities of conducting fieldwork at major pilgrimage events, characterised by excessive populations of participants, limited infrastructure and varying levels of organisation, pose significant challenges (Abdulredha et al., 2018). For example, the Arba’een event lasts up to 40 days, with pilgrimage taking place 24/7, implying the prolonged and highly laborious nature of empirical data collection. Thirdly, the focus of existing research on large-scale events often prioritises organisational aspects (for instance, the challenges of volunteer selection and training) (Cuskelly et al., 2021) and safety/security concerns (e.g. the use of illegal drugs) (Zolopa et al., 2021), with environmental considerations receiving less attention (Pereira et al., 2017). This is often, again, because of perceived difficulties associated with the fieldwork’s logistics and on-site data collection (Mohamad et al., 2012). Lastly, the interdisciplinary nature of such research, requiring expertise in environmental science, events management, religious studies, hospitality management and social sciences (Filimonau et al., 2025), may hinder its development. Addressing this research gap is crucial for developing waste management strategies that are more environmentally sustainable and culturally sensitive.

The critical review of existing literature on solid waste management in the context of religious festivals, as provided above, reveals a two-fold gap: (1) a scarcity of robust, in-situ empirical data on the quantitative scale and composition of food and plastic waste generated during major religious events, and (2) a limited understanding of the complex socio-cultural and behavioural drivers that underpin these waste streams from multi-stakeholder perspectives. Addressing these gaps necessitates a holistic approach. This is because a waste audit, while providing detailed quantitative estimates of solid waste, does not always inform the underlying reasons for its occurrence or enable the identification of key actors to be involved in its management. Therefore, a mixed-methods research design, integrating waste audits to quantify the problem’s magnitude with semi-structured interviews to uncover the stakeholder perceptions, is essential. This dual approach allows for a holistic investigation, providing answers to the ‘what’, ‘how’ and the ‘why’ of solid waste generation in the unique context of religious pilgrimages, thus reinforcing the scientific rigor and interdisciplinarity of analysis.

Accordingly, this study has set to analyse the dynamics of food and plastic waste generation during the 2023 Arba’een pilgrimage in Karbala, Iraq. It seeks to answer the following research questions: (1) What is the magnitude of food and plastic waste generated during the 2023 Arba’een pilgrimage? (2) What are the key socio-cultural and managerial factors influencing waste generation and management? (3) What are the perspectives of main stakeholders (i.e. pilgrims, mawkib owners/operators, religious leaders and municipal authorities) on solid waste and its management? To this end, employing a mixed-methods approach, the study will quantify the magnitude of food and plastic waste generated across selected mawkibs and municipal solid waste collection sites. To explore the drivers of waste generation and potential approaches to waste minimisation, the study will supplement these quantitative datasets with qualitative data obtained through in-depth, semi-structured interviews conducted with pilgrims, mawkib owners/operators, municipal authorities and religious leaders. By integrating waste measurements with interview insights, this study strives to provide a more nuanced understanding of the complex dynamics of food and plastic waste generation and management within the unique context of a major religious festival.

The study makes the following contributions to knowledge. Firstly, it provides empirical data on the scale of food and plastic waste generated during a major religious festival, the Arba’een pilgrimage. Past studies conducted on this topic to date collected data almost 10 years ago (Abdulredha et al., 2017) and after the event was completed (Abdulredha et al., 2020). Furthermore, these studies relied on food and plastic waste data self-reported by accommodation providers and pilgrims rather than direct in-situ measurements (Abdulredha et al., 2018). Although these analytical insights are valuable, they do not necessarily capture the dynamics of waste generation *during* the event. Their reliance on self-reported data may have also been subjected to biases, such as social desirability, that are widespread in research on such sensitive business and societal issues as food and plastic waste generation (Kormos and Gifford, 2014). Most importantly, extant research does not illuminate the drivers of food and plastic waste generation by pilgrims. Accordingly, this current study adds to knowledge by evaluating the socio-cultural and logistical factors that influence generation and management of food and plastic residues at a large-scale religious event, thus providing insights beyond waste quantification. Lastly, by using the case of the Arba’een pilgrimage, the study expands an emerging academic discourse on the need for broader understanding of waste management opportunities and challenges during large-scale religious events (Shinde, 2021).

From the practical perspective, the study will provide empirical data on food and plastic waste generation during the Arba’een pilgrimage to inform the design of targeted mitigation interventions. Besides, the qualitative insights obtained in this study can make these interventions more culturally sensitive and respectful of religious traditions while promoting environmental sustainability. Lastly, it is anticipated that the study’s results can support municipal authorities in Karbala and other host cities in Iraq and globally in improving waste collection and disposal infrastructure, thereby minimising the environmental impact of religious pilgrimages and/or other large-scale events.

## Materials and methods

### Study context

This study investigated the generation of food and plastic waste during the Arba’een pilgrimage in Iraq, focusing on the event held in 2023 ending on 4th and 5th of September. The Arba’een pilgrimage is one of the world’s largest annual religious festivals, culminating in Karbala and involving millions of pilgrims travelling primarily between the cities of Najaf and Karbala (Aljazeera, 2023). These pilgrims come from not only within Iraq but also neighbouring countries, including Iran, Azerbaijan and Saudi Arabia. In 2023, 22,019,146 pilgrims attended the festival (Al-Abbas Holy Shrine, 2023).

A unique and significant feature of the pilgrimage is the presence of numerous volunteer-run service stations, commonly called mawkibs, providing food and water, and sometimes lodging, small repairs and mobile phone charging docks, to pilgrims (Aljazeera, 2023). Such stations can be found in solid structures, such as buildings located on the pilgrimage route, but they can also be temporarily set, such as in makeshift tents and fabric shelters, to accommodate considerable demand during the event. Mawkibs provide their services free of charge as a symbol of hospitality and generosity (Aljazeera, 2023). It is not unusual for the owners of mawkibs to compete against each other in terms of the quality and quantity of the food they provide to gain appreciation, respect and positive word-of-mouth (Movahed et al., 2024).

During the 2023 pilgrimage, the total number of registered/licensed mawkibs was 12,750 (Al-Abbas Holy Shrine, 2023). It is important to note that some mawkibs operate without registration/license; these are usually represented by small food stalls managed by families residing on the main road to Karbala. Because these mawkibs have no license to operate, they are not accounted for in the pilgrimage statistics. The number of such illegal mawkibs is however deemed high with estimates suggesting numbers exceeding the number of official operators (Al-Abbas Holy Shrine, 2023).

An average mawkib recruits between 5 and 30 individuals, usually volunteers, who serve pilgrims every day, 24/7, taking turns if necessary. The busiest part of the pilgrimage is the last 8–10 days of the festival (Aljazeera, 2023). However, most mawkibs choose to stay open for around 20 days to demonstrate hospitality and look after the pilgrims well (Al-Abbas Holy Shrine, 2023). It is estimated that, in 2023, the cost of food served by registered mawkibs was circa USD 200 million (Al-Abbas Holy Shrine, 2023). It is also estimated that, during the 2022 event, licensed mawkibs distributed circa 4.5 million meals and 20 million bottles of water in Karbala and along the main routes leading to it (Al-Abbas Holy Shrine, 2022).

Based on their size, registered mawkibs can be divided into three categories: small, medium and large. Small mawkibs are represented by makeshift tents or temporary fabric structures; they are served by 3–7 volunteers, and their serving capacity is limited to 15 people at once. As an indicator of its capacity, a ‘typical’ small mawkib can provide about 5000 water bottles or plastic cups of water per day (Aljazeera, 2023). Medium mawkibs can operate in makeshift structures, but they can also be located in buildings. They are served by 8–15 volunteers and can accommodate 15–30 pilgrims at once. Lastly, large mawkibs operate in buildings, often of significant size. They recruit over 15 volunteers and have the capacity to serve over 30 pilgrims at once. In 2023, 60%, or 7650, registered mawkibs represented the ‘small’ category; 20%, or 2550, were ‘medium’ and 20%, or 2550, were ‘large’ (Al-Abbas Holy Shrine, 2023).

A mixed-methods approach was employed, combining food and plastic waste audit (quantitative part) with stakeholder interviews (qualitative part). This approach aimed to quantify waste generation at key points and explore the underlying causes, attitudes and management challenges associated with food and plastic waste occurrence during the pilgrimage. This enabled data triangulation which is crucial for empirical research on solid waste generation and management to obtain more accurate and reliable results (Yuxi et al., 2023).

### Waste audit

Quantitative data on food and plastic waste were collected daily for 20 days to account for the main pilgrimage period, that is, starting on 12th August and finishing on 5th September 2023. Data collection occurred at two key locations, that is, (1) selected mawkibs along the main pilgrimage route; and (2) municipal waste collection points in Karbala where food and plastic waste generated outside of mawkibs was collected for subsequent disposal.

#### Mawkib waste audit

A total of 18 mawkibs located along the Najaf–Karbala route were included in the sample. A stratified sampling strategy was used, categorising mawkibs based on their estimated serving capacity and representative share of the total number of mawkibs with a license to operate during the pilgrimage, as explained above, that is, small (*n* = 10), medium (*n* = 4) and large (*n* = 4). The selection process within each stratum involved convenience sampling based on accessibility and consent. To recruit willing participants, the research team approached multiple mawkib owners 1–2 days before the start of pilgrimage, at the stage of opening (large- and some medium-sized mawkibs) and setting up (small- and some medium-sized mawkibs). When recruiting willing participants, the researchers explained the aim of the study and requested a consent to be on-site during the pilgrimage for auding waste. Recruitment was extremely difficult because most mawkib owners declined to participate in fear of the negative effect that this study’s findings could have on their reputation. This was despite repeated reassurance of complete data confidentiality and anonymity of the study’s findings. In total, the research team approached 102 mawkibs before securing consent from 18 participants, thus recording a response rate of circa 20%. No incentives were provided to the mawkib owners for participation.

It is important to note that this study only recruited mawkibs with a license to operate. An attempt was made to also engage illegal mawkibs; however, their recruitment failed. Informal conversations with owners of such mawkibs revealed that they did not want to collaborate with the research team fearing that this study’s findings could damage their business due to non-compliance with the event organisers’ demand for licencing. This exclusion constitutes a significant methodological limitation, as these unregistered operators are estimated to be numerous, potentially equalling or exceeding licensed mawkibs in number (Al-Abbas Holy Shrine, 2023). It is possible that these informal mawkibs differ significantly in their waste generation profile and handling. Anecdotal evidence attributed to observations made by the research team during fieldwork suggests that illegal mawkibs may rely more heavily on basic, readily available food items, utilise simpler and potentially more disposable packaging materials and often operate with less formal waste management practices, leading to a higher propensity for littering rather than depositing waste at official municipal waste collection points. Consequently, the waste figures presented in this study, while deemed sufficiently robust for the sampled population, should be considered conservative estimates, representing an underestimation of the total waste generated during the pilgrimage. Future research should critically explore innovative and culturally sensitive approaches to integrate informal operators in solid waste audits, potentially through collaborative partnerships with community leaders who may represent ‘gate keepers’, ethnographic observational techniques, or even advanced remote sensing. Such research can map broader waste/litter generation patterns, thus providing a more comprehensive and accurate understanding of the pilgrimage's food and plastic waste footprint.

At each recruited mawkib, food and plastic waste audit took place throughout each pilgrimage day within the study period of 20 days. For procedural integrity and data reliability, research team members and specially trained research assistants represented by student volunteers were deployed. Prior to fieldwork, all student research assistants underwent an intensive 2-day training programme. This training covered detailed waste identification and segregation protocols, such as distinguishing food types and plastic categories, precise weighing techniques using portable digital scales (see next paragraph) and adherence to ethical guidelines, including cultural sensitivity during on-site observation. Inter-observer reliability was established through joint practice sessions and initial test-runs at a dedicated site represented by a tent resembling a small mawkib. Here, measurements were independently taken by multiple observers and subsequently compared, with any discrepancies resolved through calibration exercises supervised by the research team members until a consistent measurement protocol was achieved. During the 20-day audit, team members operated in staggered 8-hour shifts to ensure continuous 24-hour coverage and mitigate potential fatigue bias, thereby maintaining consistent data quality throughout the study period. Furthermore, a pilot study was conducted for 1 day prior to the start of the pilgrimage at one typical volunteer mawkib to test the research design, refine data insertion sheets and troubleshoot potential logistical challenges before the main data collection commenced.

Throughout a full 24-hour cycle, the team observed the process of providing and consuming food and beverages by pilgrims. The mawkib owners deposited any food leftovers and plastic waste generated by pilgrims in refuse bags. When full, these bags were carefully inspected by the researchers and measurements of food and plastic residues were made by the method of direct weighing, as recommended by Wang et al. (2017). This method was selected because it is considered more objective and accurate than the method of self-reports with its well-recognised issues of under-estimates and poor recall (Qian et al., 2022).

Portable digital scales measuring weight in kg were utilised for precision of the audit. The food and plastic waste fractions were separated prior to weighing to avoid cross contamination. Precise compositional analysis of food and plastic waste was not undertaken due to logistical and time constraints. However, it was observed by the researchers that unconsumed rice, legumes and bread were dominant in food leftovers, whereas polyethylene terephthalate (PET) bottles, polystyrene (PS) cutlery and low-density polyethylene (LDPE) bags were prevalent in plastic residues. Supplemental material, Appendix 1, demonstrates examples of food and plastic waste generated during the event.

Following completion of waste audits, the total weight of food and plastic waste generated by each surveyed mawkib was calculated. Food and plastic waste were then averaged per mawkib in each respective category. Table 1 presents the results of waste audit.

**Table 1.** Results of food and plastic waste audit in mawkibs (*n* = 18).

Mawkib category	Mawkib ID	Food waste			Plastic waste		
		Total, kg, 20 days	kg, per day	Per average mawkib in this category, kg per day	Total, kg, 20 days	kg, per day	Per average mawkib in this category, kg per day
Small	1	350	17.5	12.77 ± 4.77	140	7	6.13 ± 3.35
	2	196	9.8		98	4.9	
	3	316	15.8		189.6	9.48	
	4	280	14		140	7	
	5	240	12		120	6	
	6	270	13.5		135	6.75	
	7	210	10.5		126	6.3	
	8	272	13.6		84	4.2	
	9	260	13		130	6.5	
	10	160	8		64	3.2	
Medium	11	560	28	33.68 ± 10.7	224	11.2	15.13 ± 6.46
	12	864	43.2		432	21.6	
	13	810	40.5		324	16.2	
	14	460	23		230	11.5	
Large	15	1760	88	75.5 ± 12.5	880	44	39.4 ± 7.4
	16	1680	84		840	42	
	17	1320	66		792	39.6	
	18	1280	64		640	32	

The results demonstrate that, daily, an average mawkib from a ‘small’ category generated 12.77 ± 4.77 kg of food waste and 6.13 ± 3.35 kg of plastic waste. An average ‘medium’ mawkib produced 33.68 ± 10.7 and 15.13 ± 6.46 kg of food and plastic waste per day, respectively. An average mawkib from a ‘large’ category generated 75.5 ± 12.5 kg of food waste and 39.4 ± 7.4 kg of plastic waste, daily. These variations in the weight of waste produced across mawkibs are well aligned with their capacity to serve food. Importantly, the intra-category variance is the largest for small mawkibs, which can be explained by their locations (i.e. further from the centre) and specialism; for instance, some small mawkibs serve food which can be described as ‘basic’, such as rice and bread, whereas some provide more sophisticated dishes, such as meat and fish. Likewise, while some mawkibs provide bottled water which can be taken away from the pilgrims and disposed of later, some stations offer water refills on site, thus having more plastic residues generated in situ. Interestingly, the intra-category variance for large mawkibs is the smallest as it does not exceed 20%. This can be attributed to their more consistent specialism, that is, they provide a similar variety of meals and beverages.

To estimate food and plastic waste for all mawkibs, the average figures from Table 1 were extrapolated by multiplying them with the number of mawkibs from each category, as recommended by Leverenz et al. (2021). Although extrapolation results should be taken with caution, they are preferred in situations where surveying large populations for waste audit is unfeasible (Filimonau et al., 2019), such as in the case of mawkibs in the current study. The extrapolation results are presented in Table 2.

**Table 2.** Extrapolation results, food and plastic waste generated in mawkibs.

Mawkib category	Total number of mawkibs with a licence to operate	Average food waste per mawkib, kg	Total food waste, per mawkib category, kg	Average plastic waste per mawkib, kg	Total plastic waste, per mawkib category, kg
Small	7650	12.77 ± 4.77	1,953,810 ± 36,490	6.13 ± 3.35	938,349 ± 25,630
Medium	2550	33.68 ± 10.7	1,717,425 ± 27290	15.13 ± 6.46	771,375 ± 16,470
Large	2550	75.5 ± 12.5	3,850,500 ± 31,880	39.4 ± 7.4	2,009,400 ± 18,870
Total	12,750	–	7,521,735 ± 55,610	–	3,719,124 ± 35,840

The standard deviation for the total extrapolated waste within each mawkib category (small, medium and large) was calculated by multiplying the standard deviation of the average daily waste per mawkib in that category by the total number of mawkibs in that category. To determine the standard deviation of the overall total food waste and plastic waste across all mawkib categories, independence between the categories was assumed. This is because, during the pilgrimage, mawkibs operated largely independently in their waste management practices. Under this assumption, the variance of the overall total is the sum of the variances of the total extrapolated waste for each category. Therefore, the standard deviation of the total extrapolated waste for each category was squared to obtain the variance, then these variances across the three categories were summed, and the square root of this sum was taken to obtain the standard deviation of the overall total.

The results demonstrate that, when taken together, within 20 days of the pilgrimage, small, medium and large mawkibs may have generated 1953.8 ± 36.49, 1717.4 ± 27.29 and 3850.5 ± 31.88 tonnes of food waste, respectively. The total weight of food wasted in all mawkibs may have reached 7521.7 ± 55.61 tonnes. As for plastic waste, small, medium and large mawkibs may have generated 938.4 ± 25.63, 771.4 ± 16.47 and 2009.4 ± 18.87 tonnes, respectively, amounting to 3719.2 ± 35.84 tonnes all together. Interestingly, large mawkibs produced over 50% of all food and plastic residues. Additionally, small mawkibs generated over 25% of food and plastic waste. The primary share of large mawkibs in food and plastic waste generation across all mawkib categories may have been potentially due to the considerable amounts of waste each of them produced in the result of serving large numbers of pilgrims. The second position of small mawkibs in waste generation can be explained by their prevalence in the studied population where they represented 60% of the total number of licensed stations during the 2023 pilgrimage event.

#### Municipal disposal container audit

During the festival, the researchers witnessed that some pilgrims would take food and beverages away from mawkibs for later consumption. For example, it was often the case during busy times when a considerable number of pilgrims would queue for food and drinks. In such cases, some pilgrims would choose to take the supplies away and consume them off site. Therefore, to account for this extra wastage, it was decided to supplement figures on food and plastic waste obtained by an in-situ audit, as described above, with data from other sources.

To this end, an audit of municipal collection containers located alongside the pilgrimage route in Karbala was made. These containers were placed in different parts of the route specifically to collect waste generated by pilgrims outside of mawkibs. In total, there were 10 containers with the capacity of 2 tonnes each. The containers were collected once a day during the first 10 days of the festival and twice a day during the last 10 days. The waste collected from these containers was disposed of by landfilling.

The containers were audited each day during the festival prior to their collection for disposal. The assessment of waste composition within these containers was performed by members of the research team and trained student research assistants. Given the substantial volume and mixed nature of the waste within these large containers, as recommended by Hanks et al. (2014), a visual estimation method was employed to determine the proportions of major waste fractions for each container’s contents. Besides, for quality assurance and to enable inter-observer consistency in visual estimates (Filimonau et al., 2022a), a random subset of 20% of the containers was independently assessed by two different research team members, and their estimations were cross-referenced. This systematic visual audit revealed that, on average, 60% of the waste was represented by organic fraction (predominantly food waste) and 40% was attributed to plastic residues. The total weight of food waste generated during the event from these containers was calculated as 360 tonnes. Plastic waste accounted for 240 tonnes.

#### Aggregate waste figures

By combining the results of waste audits undertaken in mawkibs and municipal collection containers, it can be concluded that the total weight of food waste generated during the 2023 pilgrimage amounted to 7881.7 tonnes. Plastic waste weighed, in total, 3959.1 tonnes. As 22,019,146 pilgrims attended the festival in 2023 (Al-Abbas Holy Shrine, 2023), it can be concluded that food waste generation per pilgrim was equal to 0.36 kg and plastic waste – 0.18 kg. It can also be concluded that the amount of food waste generated daily during the pilgrimage equated 376 tonnes, and plastic waste – 198 tonnes.

It should be noted, however, that while these food and plastic waste figures provide novel insights into solid waste generation during the 2023 Arba’een pilgrimage, it is crucial to interpret them within the specific context of this event, rather than generalise them towards other religious festivals. This is discussed further in this study’s limitations.

### Qualitative data collection

Semi-structured interviews were conducted to explore stakeholder perspectives, triangulate quantitative findings and understand the drivers of waste generation during the festival. Participants were recruited using a combination of convenience, quota and purposive/expert sampling to ensure the representation of different roles and experiences within the sample. Mawkib owners/operators (*n* = 20), representing service providers of varying sizes, were recruited by convenience sampling given substantial difficulties associated with their engagement in this study, as explained in section ‘Mawkib waste audit’. Although convenience sampling was necessary for mawkib owners/operators due to recruitment challenges, it is acknowledged that this method may have introduced potential biases towards mawkibs that were more accessible or whose owners were more willing to participate. Pilgrims (*n* = 36), representing the consumers of mawkib services and primary waste generators, were recruited via quota sampling based on gender, age and ethnicity, aiming to reflect the estimated proportions within the pilgrim population based on available demographic information from previous pilgrimages (Almerja, 2023). Lastly, municipal solid waste management officials (*n* = 2) from the Karbala municipality, involved in formal waste collection and disposal, and religious leaders (*n* = 2) from Karbala known for their influence during the pilgrimage, were recruited via purposive/expert sampling. Municipal solid waste management officials were selected based on their senior roles and direct involvement in waste collection and disposal operations during the Arba’een pilgrimage. Religious leaders were chosen based on their recognised influence within the Karbala community and their known engagement with issues related to pilgrim conduct during the festival. Table 3 lists this study’s participants.

**Table 3.** Interview participants (*n* = 60).

Participant ID	Gender	Age	Participant ID	Gender	Age
P1	Male	In their 30s	P31	Female	In their 40s
P2	Male	In their 50s	P32	Female	In their 30s
P3	Male	In their 50s	P33	Male	In their 50s
P4	Male	In their 30s	P34	Male	In their 30s
P5	Female	In their 40s	P35	Female	In their 40s
P6	Female	In their 40s	P36	Female	In their 40s
P7	Male	In their 40s	M1	Male	In their 40s
P8	Male	In their 40s	M2	Male	In their 40s
P9	Female	In their 30s	M3	Male	In their 40s
P10	Female	In their 30s	M4	Male	In their 40s
P11	Female	In their 40s	M5	Male	In their 30s
P12	Female	In their 30s	M6	Male	In their 60s
P13	Female	In their 30s	M7	Male	In their 30s
P14	Male	In their 50s	M8	Male	In their 50s
P15	Male	In their 50s	M9	Male	In their 30s
P16	Male	In their 30s	M10	Male	In their 60s
P17	Male	In their 60s	M11	Male	In their 60s
P18	Female	In their 30s	M12	Male	In their 50s
P19	Female	In their 40s	M13	Male	In their 40s
P20	Female	In their 40s	M14	Male	In their 30s
P21	Male	In their 40s	M15	Male	In their 60s
P22	Male	In their 30s	M16	Male	In their 60s
P23	Male	In their 50s	M17	Male	In their 30s
P24	Female	In their 40s	M18	Male	In their 40s
P25	Female	In their 40s	M19	Male	In their 40s
P26	Male	In their 60s	M20	Male	In their 40s
P27	Male	In their 60s	A1	Male	In their 50s
P28	Male	In their 40s	A2	Male	In their 40s
P29	Male	In their 40s	R1	Male	In their 60s
P30	Male	In their 60s	R2	Male	In their 50s

P: pilgrim; M: mawkib owner/operator; A: municipal authority representative; R: religious leader.

Interviews explored the following topics: (1) perceptions of the scale of food and plastic waste generated during the event; (2) perceived realism of waste estimates; (3) primary reasons for food wastage (e.g. over-serving, spoilage, mawkib owner/operator and pilgrim behaviour); (4) rationale for plastic use (e.g. hygiene, cost, convenience); (5) waste disposal practices and awareness; (6) challenges faced in managing waste (from mawkib, pilgrim and municipal perspectives); (7) religious perspectives on waste versus hospitality (from pilgrim and religious leaders’ perspective) and (8) suggestions for waste minimisation. Interview guides (main questions) are provided in Supplemental material, Appendix 2. These guides were developed in English and back translated in Arabic. Prior to fieldwork, interview guides were piloted with two volunteers representing each of the focal stakeholder categories. The guides were also checked for content and face validity with three academics majoring in religious studies, hospitality management and waste management.

Interviews were conducted in Arabic and lasted between 35 and 76 minutes providing a total of over 1900 minutes of data. Interviewing took place on different dates of the festival and in different locations to ensure comfort of participants. Due to their availability, interviews with municipal solid waste management officials and religious leaders were conducted shortly after the event, that is, in the last two weeks of September 2023. Interviews were digitally recorded with participant consent, transcribed verbatim and professionally translated for subsequent analysis. Participation in interviews was not incentivised.

Interview transcripts were analysed thematically. An inductive approach was used (Clarke and Braun, 2017) to identify recurring themes, patterns, attitudes and explanations related to food and plastic waste generation and management. Coding was undertaken independently by two members of the research team. Any discrepancies in the initial coding were discussed and resolved through consensus to ensure consistency and enhance the reliability of the identified themes. NVivo 14 (https://lumivero.com/products/nvivo/) was utilised to visualise and connect the codes.

### Ethical considerations

This study received full research ethics approval from the University’s research ethics committee of the lead researcher. Aligned with this approval, rigorous measures were implemented to ensure the informed consent, anonymity and confidentiality of all participants throughout the research process. These measures were critical given the cultural and reputational sensitivities around data misuse, particularly concerning mawkib owners and their operations. Prior to their participation, all individuals (mawkib owners/operators, pilgrims, municipal officials and religious leaders) received a comprehensive oral explanation of the study’s purpose, the nature of their involvement, the voluntary nature of participation and their right to withdraw at any time without negative consequence. Written informed consent was obtained from all participants prior to data collection. For several individuals who were unable to read or write, verbal consent was obtained in the presence of an impartial witness.

For anonymity and confidentiality, all collected data were de-identified. Personal identifiers, such as names, direct identifying locations or specific organisational names, were removed from interview transcripts and replaced with unique participant codes. All audio recordings were securely stored on password-protected, encrypted university servers and were deleted after transcription. Access to the raw data was restricted to the core research team members. No identifiable information was shared with any third parties. Lastly, the core research team alongside the research student assistants underwent a dedicated training on local customs and respectful engagement protocols to ensure the study was conducted in a culturally sensitive manner throughout the fieldwork.

## Results and discussion

### Waste audit

The results demonstrate that a major pilgrimage event, such as the Arba’een festival, generates disproportionate amounts of food and plastic residues within a relatively short period of time, that is, 20 days. As for food waste, UNEP (2024) suggests that 1.1 Mt of food is wasted in the Iraqi sector of hospitality and foodservice provision per year, or an equivalent of 23.4 kg per resident. This suggests that an average Iraqi wastes 0.064 kg of food daily. The results of the current study indicate that an average pilgrim wastes 0.36 kg which is over five times more than an average Iraqi resident producing per day. The results also show that the Arba’een festival may have produced 0.72% of total food waste generated in the Iraqi sector of hospitality and foodservice provision per year.

As for plastic waste, World Population Review (2025) posited that circa 684,000 tonnes of plastic waste is produced across all sectors in Iraq per year, or an equivalent of 14.5 kg per resident. This suggests that an average Iraqi generates 0.04 kg of plastic waste daily. The current study demonstrates that an average pilgrim produces 0.18 kg of plastic waste which is over four times more than an average Iraqi resident producing per day. The results also show that the Arba’een festival may have produced 0.58% of total plastic waste generated in Iraq per year. World Population Review (2025) further argued that over 85% of plastic waste generated in Iraq is mismanaged as it is disposed of by such methods as illegal dumping and landfilling. This showcases the challenge presented by the Arba’een festival which produces considerable amounts of plastic residues in presence of poorly managed disposal system.

Lastly, Abdulredha et al. (2020a) showed that another major religious festival in Iraq, Ashura, attended by up to 3.5 million pilgrims annually, generates 284 tonnes of solid waste per day. This current study demonstrates that, when combined, food and plastic waste produced during the Arba’een festival may have amounted to 574 tonnes daily or two times more than that during Ashura. Furthermore, Abdulredha et al. (2018) estimated that an average guest staying in a hotel in Karbala during the pilgrimage generated 0.89 kg of solid waste per day. This current study provides a lower estimate of 0.54 kg of (food and plastic combined) waste per pilgrim. However, it is important to note that Abdulredha et al. (2018) estimated solid waste generated by hotel guests rather than pilgrims, which included waste produced by support services, such as breakfast and evening meals. The higher figure reported in their study can be attributed to the fact that hotels, especially upmarket and luxury, waste more food than an average resident, see Kasavan et al. (2022) for a review. Therefore, the figure obtained in the current study may represent a more accurate and up-to-date estimate of the amount of food and plastic residues generated during the Arba’een festival. Coupled with underdeveloped solid waste management infrastructure in the city of Karbala (Abdulredha et al., 2020b), the results of the current study suggest that solid waste presents a major challenge to the municipal authorities in Karbala who have to deal with significant amounts of food and plastic residues occurring within a short time.

It is important to note, however, that although this study provides the first empirical estimates of food and plastic waste generation during the Arba’een pilgrimage, it is necessary to acknowledge that these figures likely represent an underestimation of the true total waste. This is because, as outlined in the methodology, the data collection was limited to licensed mawkibs. As the number of illegal mawkibs is estimated in Al-Abbas Holy Shrine (2023) can equal or even exceed the number of legal, official operators, and because these illegal mawkibs are more likely to operate with less formal solid waste management practices (e.g. they may be reluctant to deposit food and plastic waste generated on their premises to municipal collection points and disperse it by littering instead), their unmeasured contribution means the overall waste generation during the pilgrimage can double the figures obtained in the current study. Importantly, this extra wastage alongside its highly likely mismanagement, as per above, further exacerbates the already substantial logistical challenge of food and plastic waste management for municipal authorities.

Another important point to note is that environmental conditions linked to the pilgrimage season are likely to play a significant, but overlooked (due to fieldwork logistics and organisation reasons), role in exacerbating food waste. The Arba’een pilgrimage takes place during late summer and early autumn months, which are characterised by high ambient temperatures. Heat drastically accelerates the perishability of cooked meals, fresh produce and other perishable food items (Aung and Chang, 2014). In the absence of extensive and reliable cold chain infrastructure for all mawkibs, especially the smaller, temporary ones, food spoilage due to rapid degradation becomes inevitable, contributing substantially to the overall volume of food waste. This climatic factor adds a critical dimension to the waste generation challenge, distinct from behavioural or logistical factors, emphasising the need for robust food management strategies adapted to environmental realities, especially to minimise waste caused by these conditions. This climatic factor represents one of the limitations of the current study as it could have affected the amounts of food waste generated and its management during the festival.

### Interviews

The interview findings reveal that mawkib owners/operators and pilgrims blame each other for excessive waste generation. The tendency for mawkib owners/operators to attribute food and plastic waste primarily to pilgrim behaviour reflects their direct experience with the consumption patterns they observe: ‘*Pilgrims are greedy. They come, take whatever they see, bite a bit, and put the rest in the bin. Then, they go to another stall and do exactly the same. Greediness is the main issue here’* (M5). Having invested significant resources and effort in providing generous hospitality, mawkib owners/operators may witness pilgrims taking more food than they can consume, discarding significant portions or being unmindful of waste generation. This suggests that the sheer volume of waste is perceived as a direct consequence of individual pilgrim choices: ‘*We [mawkibs] provide food as a matter of generosity. They [pilgrims] don’t appreciate it which is so sad to see because it’s a religious walk and wasting food is haram is Islam’* (M12).

Furthermore, given the focus on upholding traditions of hospitality, mawkib owners/operators may (in)advertently downplay the impact of their own practices, such as portion sizes or the reliance on disposable items, in contributing to the overall waste stream: *‘It’s a major event, so we need to show off our hospitality, even if it comes at a cost of food waste. It’s better to waste food than to waste reputation’* (M18). This perspective is often reinforced by the scale of the event, where the large number of pilgrims can make individual consumption habits appear to be the dominant factor.

Conversely, pilgrims often frame mawkib practices as significant contributors to wastage: *‘The portions are too big, in my opinion. There’s no need to serve mounts of rice, it’s so hot, people are not that hungry anyway. Besides, it’s pilgrimage, we must be modest’* (P2). Pilgrims may perceive the competitive drive among mawkibs to offer abundant quantities of food as leading to excessive portion sizes that are simply impossible to finish, thus inevitably resulting in food waste: ‘*They shout ‘come here, come here, best rice in town, the largest portion in town’, so people rush to them. . . There’s no need for this, really, all we need is some food to keep us going and a lot of water to sustain the walk’* (P21).

Furthermore, the widespread use of single-use plastics by mawkibs for serving food and beverages is a visible and tangible source of plastic waste, leading pilgrims to see mawkib owners/operators as directly responsible for its generation: ‘*Some pour water, but some give us [water] bottles. The latter are more convenient, so most pilgrims take them and then dump them off’* (P13). Pilgrims also argue that the burden of providing adequate waste management infrastructure, such as sufficient and accessible bins, lies with the mawkibs as the primary providers of goods and services, or with the overarching event organisers: *‘I came from far afield. I’m tired as I’ve walked over 100 kilometres. Do you think I have the power to decide what is good for nature? No! It’s their responsibility, stall owners and the government of Karbala who organize the event’* (P19). From this viewpoint, any shortcomings in waste management are attributed to the providers rather than the consumers. This ‘blame game’ highlights a crucial gap in shared responsibility and a potential divergence in priorities, where the cultural emphasis on generous hospitality and the individual pilgrim's experience neglects considerations of environmental sustainability and waste reduction.

The blame game established in the current study can be theoretically interpreted through the lens of attribution theory (Yeboah et al., 2025), which posits that individuals seek to understand the causes of events, often attributing outcomes to either internal (dispositional) or external (situational) factors. By blaming pilgrims, mawkib owners/operators tend to shift responsibility to ‘others’ while implicitly refusing to acknowledge their own role in preventing food waste by, for instance, providing smaller food portions or offering water refills in place of single-use water bottles. Mawkib owners/operators also tend to appeal to ‘higher loyalties’, such as the Islamic traditions of hospitality, which demand guest satisfaction when justifying food waste. This enables them to ‘neutralise’ their own inaction in food and plastic waste management, the notion aligned closely with the principles of neutralisation theory in solid waste management (Coşkun and Filimonau, 2021). These patterns of attribution and neutralisation can also be observed among pilgrims as they tend to reject their own responsibility for environmentally unsustainable behaviour referring to other, situational factors beyond their control, such as large food portions, as the main causes. Again, this demonstrates the role of social attribution and various neutralisation techniques in restricting individual agency and hindering the acknowledgment of personal responsibility in waste generation.

It is important to note, however, that the blame game identified in the current study carries significant, negative, consequences for solid waste management. As shown by Koiwanit and Filimonau (2023) and Massoud et al. (2019), when responsibility is diffused or unilaterally attributed within multiple stakeholders, it can lead to inaction, as neither party feels fully empowered or obligated to comprehensively engage in waste reduction. This hinders the collective effort required to address the escalating problem of waste generation, emphasising the need for cultivating a genuine sense of shared responsibility across all stakeholder groups. As Silva and Morais (2021) argued, shared rather than diffused responsibility and mutual trust represent the determinants of successful transition to a circular economy in developing nations.

Beyond the mutual blaming between mawkib owners/operators and pilgrims, the issue of food and plastic waste during the Arba’een pilgrimage is further compounded by the perspectives of religious leaders and municipal authorities. Religious leaders interviewed frame the excessive wastage occurring during the event as a failure of both pilgrims and mawkib owners/operators to adhere to core Islamic principles, such as the prohibition of wastefulness (haram). They suggested that both pilgrims and mawkib owners/operators may, at times, act in ways that contradict Islamic teachings on the value of resources and the avoidance of extravagance. For example, for mawkib owners/operators, as religious leaders state, the principle of generosity and attention seeking (sometimes referred to as shufuni) dominates the principle of prohibition due to the unique nature of the festival: *‘It’s hard to explain this, but many people at this festival prioritise hospitality and generosity over modesty. It’s a huge event, so you should be generous, they think, as, otherwise, you’ll be seen as a bad individual. This is down to the misinterpretation of religious teachings, I guess’* (R1). This religious framing adds a moral dimension to the problem, positioning waste not only as a purely environmental or logistical challenge but also as a potential transgression of faith. This perspective of religious leaders highlights that, without a foundational shift in understanding individual and collective duties towards resource stewardship, efforts to effectively manage food and plastic waste during the festival will face difficulties and even resistance.

Similarly, municipal authorities tend to place responsibility on both mawkib owners/operators, pilgrims and, to an extent, religious leaders for the significant waste burden they create. Municipal authorities interviewed often cite logistical challenges, the sheer volume of waste generated and a perceived lack of cooperation or adherence to waste management guidelines by mawkib owners/operators and pilgrims as key obstacles in maintaining cleanliness and environmental sustainability during the pilgrimage. They also argue that religious leaders should have done a better job educating people about the need to prioritise the conservation of resources rather than showing off: ‘*The main issue is a lack of education. People don’t understand how much waste they produce and why it’s so bad for places like Karbala. . . Religious leaders should play a more active part in educating people, telling them that it’s unacceptable to waste, it’s haram, especially during the pilgrimage which should be about the appreciation of other people’s efforts, modesty and restrain’* (A2). The prevalence of this multi-directional blame, exacerbated by external factors such as budget limitations, hampers the development and implementation of integrated and effective waste management systems on the ground.

Moreover, the considerable quantities of waste generated, particularly due to the prevalence of non-biodegradable plastics and largely organic food waste, impose substantial financial strains on municipal budgets by increasing costs for collection, transportation and disposal. This economic burden, alongside the related environmental impacts, such as soil and water contamination, emphasises the urgency of improved solid waste management. It becomes especially relevant in the context of many developing nations where political and economic factors hinder the availability and efficacy of environmental conservation actions performed by local governments and municipal authorities (Bundhoo, 2018; Filimonau and Tochukwu, 2020; Koiwanit and Filimonau, 2023).

Importantly, although religious leaders and municipal authorities operate from distinct institutional frameworks and often perceive waste management responsibilities differently, as evidenced by their respective attributions of blame and focus areas as outlined in the current study, their combined influence in such country as Iraq offers a potential avenue for co-creating effective interventions. In Islam, religious leaders possess profound moral authority and, in many cases, deep community trust (Filimonau et al., 2022b). This enables them to frame waste reduction not merely as an environmental concern, but as a direct adherence to Islamic principles of stewardship, modesty and resource conservation. This spiritual legitimation is important for shaping pilgrim and mawkib behaviour during the festival, potentially overcoming behavioural barriers that secular appeals might face, such as public unacceptance of fines for littering. Concurrently, municipal authorities possess essential logistical expertise, control over waste management infrastructure, and regulatory capacity. Synergy could therefore be fostered through such joint, collaborative initiatives between religious leaders and municipal authorities as municipal support for religiously endorsed campaigns, where religious leaders actively promote messages about avoiding waste (for instance, in preachings, public addresses and digital media) with logistical backing from the municipality (e.g. providing bins, managing regular collection schedules or sponsoring educational materials that feature religious messages). Joint workshops and the co-development of guidelines on waste prevention and reduction, thus harnessing both religious values and practical waste management best practices, could broadcast a unified and, therefore, more impactful message.

Lastly, municipal authorities blamed, in private, national authorities for not providing funds to improve waste management infrastructure. This lack of funds forced them to do as much as they could to manage excessive wastage in the absence of any better solutions, such as recycling and composting facilities: *‘Look*, *we’ve been promised that a recycling plant would have been built here since 2008*. *Almost twenty years have gone now. Can you see that plant?* (A1). These external perspectives highlight that the assumed responsibility for food and plastic waste generation during the pilgrimage lies with the primary actors on the ground, albeit viewed through different lenses, that is, religious doctrine and practical management.

These findings contribute to the emerging discourse on how attribution of responsibility impacts personal attitudes towards food (Birau and Faure, 2018) and plastic (Mayer and Kohl, 2024) waste. More specifically, these findings showcase an example of the ‘blame game’ (Martin et al., 2025) occurring when multiple stakeholders attribute responsibility for wastage to one another rather than directly to themselves, thus finding scapegoats (Cherrier and Türe, 2022). The unique contribution of the current study is in demonstrating the occurrence of this blame game and scapegoating during a major religious festival where, arguably, such feeling as blame contradicts the whole nature of this event. Furthermore, this current study shows that attribution of responsibility can be observed among multiple stakeholders and not only consumers, who have been the focus of research inquiry on blame game and scapegoating to date (Shulman et al., 2021). This extends this line of scholarly discourse towards such stakeholder groups as foodservice operators, municipal authorities and religious leaders.

## Conclusion

This study provided novel, empirical insights into the complex dynamics of food and plastic waste generation during the 2023 Arba’een pilgrimage in Karbala, Iraq, one of the world’s largest annual religious festivals. Quantitative audits revealed substantial wastage, totalling 7900 tonnes of food and 4000 tonnes of plastic, with an average pilgrim generating 0.36 kg of food and 0.18 kg of plastic, collectively contributing significantly to Iraq’s annual waste figures. This considerable scale of quantified waste reinforced the thematic concerns uncovered in semi-structured interviews: a pervasive ‘blame game’ phenomenon between mawkib owners/operators and pilgrims regarding responsibility for wastage. Although mawkib owners/operators often attributed waste to pilgrim behaviour (i.e. internal attribution), pilgrims consistently cited over-provisioning and the widespread use of single-use plastics by mawkibs (i.e. external attribution), directly mirroring the large volumes and dominant fractions quantified in the waste audits. Furthermore, the large magnitude of waste audited heightened the moral tension described by religious leaders, who viewed such excess as contradicting fundamental Islamic principles of modesty and resource conservation. Concurrently, interviews revealed that high figures of food and plastic waste generated during the festival intensified the logistical and infrastructural challenges highlighted by municipal authorities, validating their calls for greater stakeholder collaboration to manage wastage. This integration of waste audit data and qualitative insights showcased how the major challenge of solid waste revealed and emphasised the perceptual, behavioural and systemic challenges in solid waste management at large-scale religious events, in Iraq and beyond.

### Practical implications

The practical implications of this study necessitate a multi-focused approach that engages each stakeholder group for more effective waste management during the Arba’een pilgrimage. Firstly, from the short-term perspective, immediate and most feasible interventions can target behavioural shifts and basic operational improvements. For mawkib owners/operators, targeted awareness and education campaigns, co-developed and co-delivered by religious authorities and event organisers, are crucial to highlight the Islamic principles against wastefulness. These campaigns could be complemented by pre-event and post-event workshops sharing practical waste reduction strategies. Religious leaders and municipal authorities can be invited to contribute to such workshops, thus showcasing their significance and ensuring better engagement. Mawkibs can also explore innovative methods for managing portion sizes, such as by offering options (e.g. *‘try and take more if you like it’*) or subtly encouraging pilgrims to take only what they need initially (for instance, by using a slogan with a religious appeal, such as *‘remember that this pilgrimage is about modesty; therefore, stay modest and only take the amount of food which you really need’*).

Secondly, mid-term interventions can build upon these steps, requiring moderate investment and coordinated stakeholder effort. Facilitating access to affordable and culturally appropriate but more sustainable alternatives to single-use plastics, such as bamboo or paper, potentially through subsidies or partnerships with manufacturers, is another key area for interventions. For example, instead of providing bottled water, mawkibs can collectively and voluntarily agree on only offering water refills and event organisers should communicate and potentially reinforce this agreement by making it a condition for licencing to mawkibs.

More specifically, providing access to economically affordable and culturally appropriate sustainable alternatives to single-use plastics represents a key area for interventions. Although transitioning from single-use plastics presents significant logistical and financial challenges due to the large scale of the pilgrimage and the volunteer-driven, donation-based nature of mawkibs, some concrete pathways can be proposed. For instance, event organisers, potentially with the aid of governmental subsidies, by finding suitable event sponsors, or through partnerships with manufacturers, could explore centralised procurement and provision of compostable cutlery, plates or bulk water dispensers to be used by mawkibs. For instance, instead of widespread distribution of single-use water bottles, as found in the current study, mawkibs could agree on offering water refills to pilgrims at designated stations along the route, with event organisers proactively communicating this change to prospective festival participants. This would however necessitate the development of a robust infrastructure of accessible refill points and, potentially, a design of a dedicated system for pilgrims to carry and store reusable bottles. On a positive side, reusable bottles could be provided for free, or for a symbolic fee, by event sponsors and carry their logo, thus aiding in promotion and raising pro-environmental awareness among pilgrims. Alternatively, a phased introduction of easily compostable alternatives, such as those made of bio-plastics, could start with specific items consumed in high volumes, such as single-use cutlery, provided there is a corresponding investment in regional composting facilities to ensure their proper end-of-life management. The development of voluntary environmental sustainability guidelines and agreements (e.g. no bottled water provided during the pilgrimage, as per above), coupled with public recognition for mawkibs implementing best practices (for instance, where the mawkibs excelling in waste reduction are named and praised by religious leaders during the event’s culminal religious services, but also where municipal authorise acknowledge waste reduction efforts by issuing ‘green’ certificates to mawkibs), can cultivate a sense of shared responsibility.

Engaging pilgrims requires comprehensive educational initiatives prior to, during, and after the pilgrimage, emphasising mindful consumption and responsible disposal of any food and plastic leftovers and residues. Clear, multilingual signage and readily accessible waste disposal infrastructure along the pilgrimage routes and in Karbala are essential, potentially supported by digital tools, such as maps and mobile phone applications, pointing towards the areas where disposal is possible but also highlighting water refill stations. The influential role of religious leaders and community figures in promoting (more) responsible waste behaviour through their own example and public endorsements can also be implemented. Like in the case of mawkibs owners/operators who have excelled in waste reduction, pilgrims can be praised for their ‘green’ actions by religious leaders during religious ceremonies.

Religious leaders themselves hold a significant opportunity to integrate environmental ethics and the Islamic imperative of resource conservation into their teachings related to the pilgrimage. Publicly endorsing sustainable practices and collaborating with environmental experts to develop religious guidance on balancing Islamic hospitality with environmental responsibility during the event can provide moral and practical frameworks to followers.

Lastly, long-term, systemic changes are essential to achieve effective solid waste management, demanding substantial financial investment and multi-level policy integration. To this end, for municipal authorities and event organisers, a key is investing in waste management infrastructure, including provision of more bins, (more) efficient bin collection services, and the exploration of recycling and composting facilities. Developing clear and culturally sensitive guidelines for mawkibs on solid waste management, coupled with free pro-environmental training for volunteers, is also important. Large-scale public awareness campaigns targeting all stakeholders are necessary to highlight the scale of the solid waste problem and encourage action. Fostering collaboration among all stakeholders and securing financial investment from national authorities for long-term infrastructure improvements are also required. By implementing these recommendations, the Arba’een pilgrimage can move towards more effective and responsible waste management practices, better aligning with religious values and the urgent need for environmental stewardship. Importantly, although these recommendations are directly informed by the 2023 Arba’een pilgrimage, their successful implementation in future years or at other religious events should require careful consideration of their specific (political, economic and cultural) contexts and potential adaptations to the actions proposed to fit these particular contexts.

### Limitations and future research directions

Despite its contributions, this study has limitations. The reliance on convenience sampling for mawkibs limits the generalisability of the quantitative findings. This also applies to the investigation of only licensed mawkibs and the exclusion of a significant number of smaller mawkibs operating illegally. The focus on waste generated by mawkibs and deposited in waste collection containers is also a limitation, as this does not consider any wastage or littering occurring illegally, such as illegal dumping in other localities and outside the main pilgrimage route. The figures of food and plastic waste obtained in the current study should therefore be considered under-estimates, and future research should aim at improving precision of measurements by extending the scope of analysis to more mawkibs and covering other localities within the pilgrimage route.

Future research should examine the impact of weather on food perishability and resultant wastage during the pilgrimage alongside approaches to be used by event organisers and mawkibs to minimise the negative effect of heat and other weather conditions on food (waste). Future research should also consider exploring longitudinal trends in waste generation, evaluating the effectiveness of targeted waste reduction interventions, examining in more detail pilgrim and mawkib attitudes and behaviour through in-depth interviews and field observations and conducting comparative analyses across different religious festivals. For instance, repeated annual waste audits conducted over multiple pilgrimage cycles/years can be arranged to track inter-annual changes in food and plastic waste generation and assess the long-term efficacy of new mitigation interventions. Evaluating the effectiveness of targeted waste reduction interventions, such as specific portion control strategies or alternative serving material trials, could benefit from quasi-experimental designs or pilot programmes with pre- and post-intervention measurements. Examining pilgrim attitudes and behaviour in more detail could involve large-scale surveys administered digitally, via smartphone apps dedicated to the pilgrimage, or via dedicated ‘Visitor information’ kiosks installed en-route, alongside in-depth ethnographic observations of food and plastic consumption and disposal practices to capture real-time choices.

Furthermore, although comprehensive steps were undertaken in the current study to ensure the rigor of the qualitative data collection and analysis, certain limitations associated with interview-based research should be acknowledged. These include the potential for interviewer bias, where researchers’ presence or their questioning style might have inadvertently influenced participant responses, particularly given the sensitive nature of discussing solid waste within religious and cultural contexts. Additionally, challenges related to the subjective interpretation of qualitative data are typical for qualitative investigations; although inter-coder reliability measures were employed to reduce its impact, the process of thematic analysis remains subject to researcher interpretation. Lastly, despite careful back-translation protocols, achieving complete linguistic and cultural equivalence can never be fully guaranteed, potentially leading to subtle translation constraints that might affect the understanding of participant expressions. These factors highlight the need for continued qualitative inquiry employing diverse methods in similar contexts to generate more reliable findings and ensure data triangulation.

The specific context of the 2023 Arba’een pilgrimage necessitates caution when drawing conclusions for other years of this festival or other religious events. The absence of detailed compositional analysis and the constraints of the 20-day data collection period also represent limitations which should be addressed in future research. Lastly, investigating the economic impacts of the pilgrimage’s waste generation also warrants further research.

## Supplemental Material

sj-docx-1-wmr-10.1177_0734242X251385955 – Supplemental material for Food and plastic waste generation at a large-scale religious festival and implications for sustainable management Supplemental material, sj-docx-1-wmr-10.1177_0734242X251385955 for Food and plastic waste generation at a large-scale religious festival and implications for sustainable management by Hana Kadum, Hussien L. Algboory, Nameer Khairullah Mohammed, Belal J. Muhialdin and Viachaslau Filimonau in Waste Management & Research
