# Supplementary material for: Food and plastic waste generation at a large-scale religious festival and implications for sustainable management
Source: Waste Manag Res. 2025 Nov 3;44(4):389–403. doi: 10.1177/0734242X251385955 (PMC12988016; doi:10.1177/0734242X251385955)
Supplement: sj-docx-1-wmr-10.1177_0734242X251385955 – Supplemental material for Food and plastic waste generation at a large-scale religious festival and implications for sustainable management [file sj-docx-1-wmr-10.1177_0734242X251385955.docx]

Appendix 1. Examples of food and plastic residues.

| 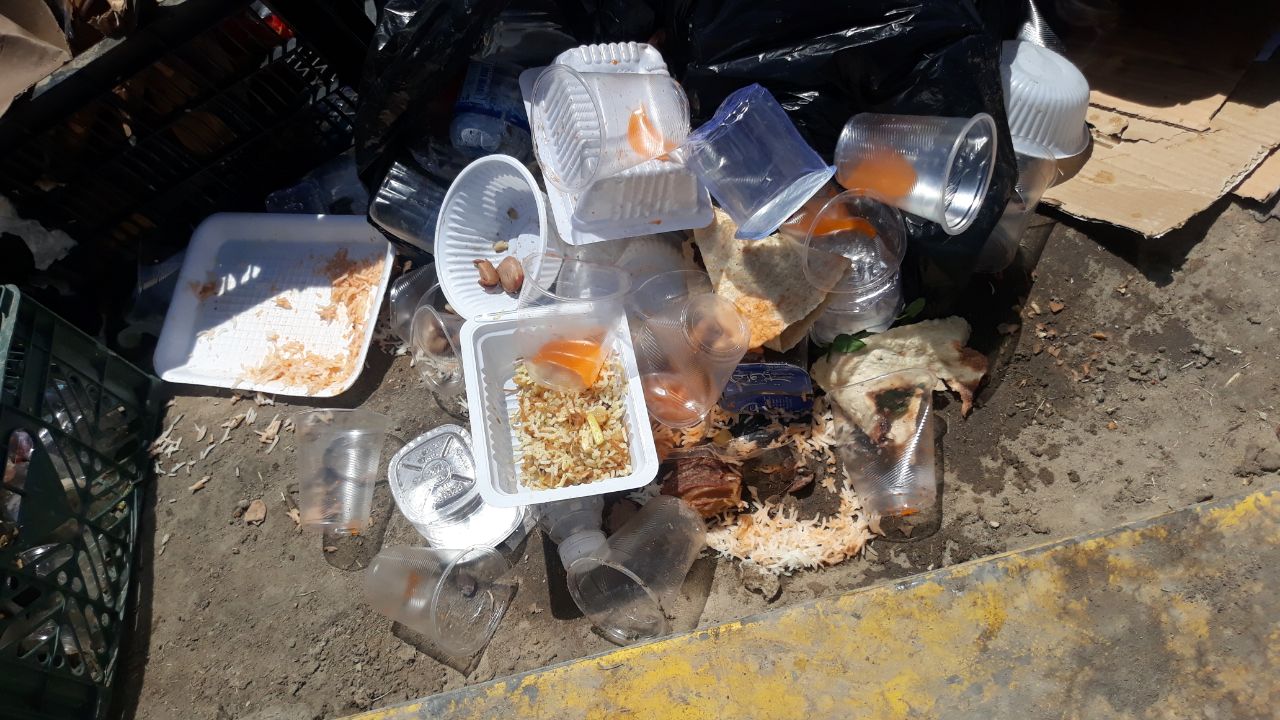 | 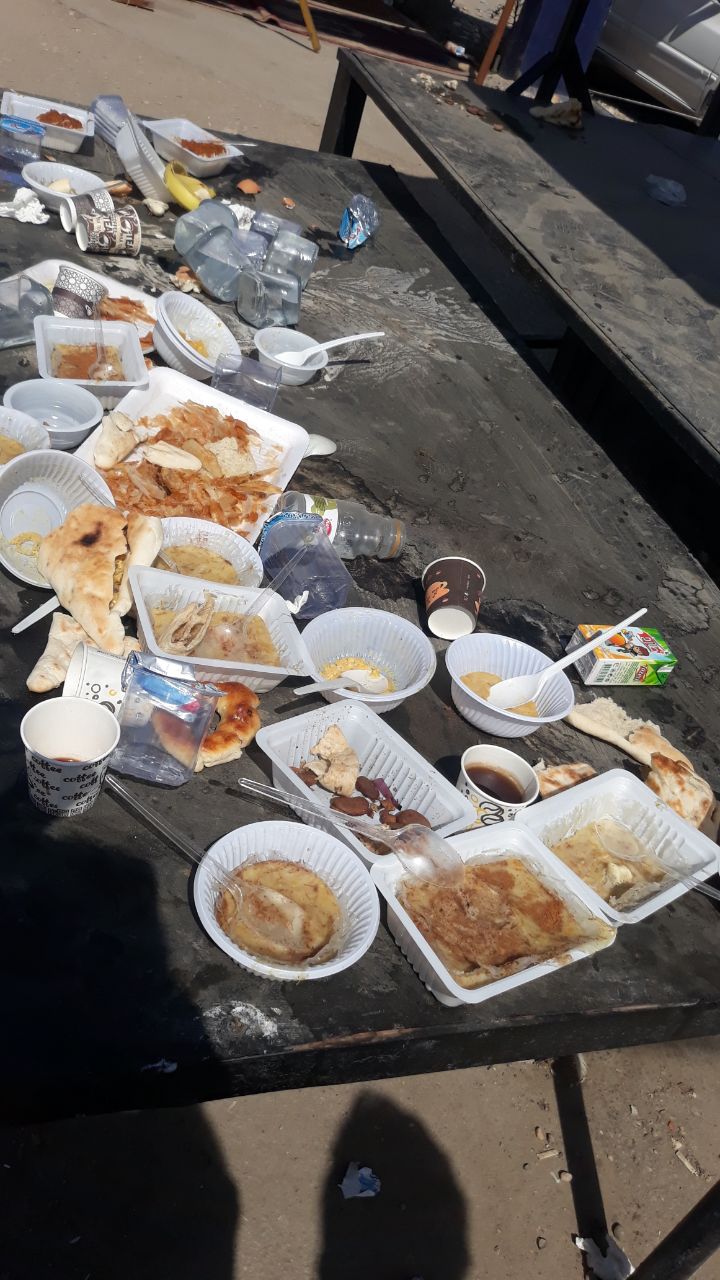 |
| --- | --- |
| 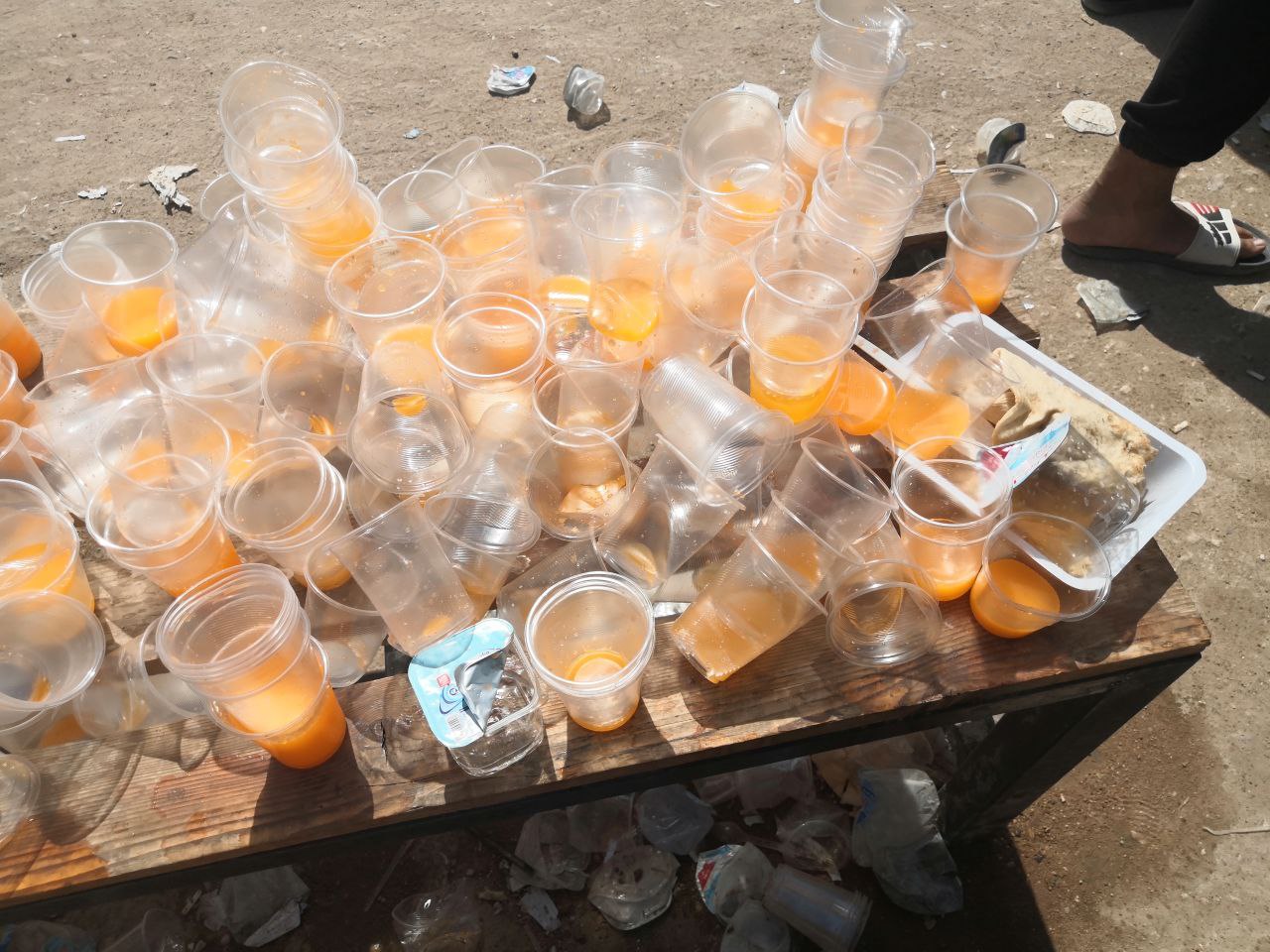 | 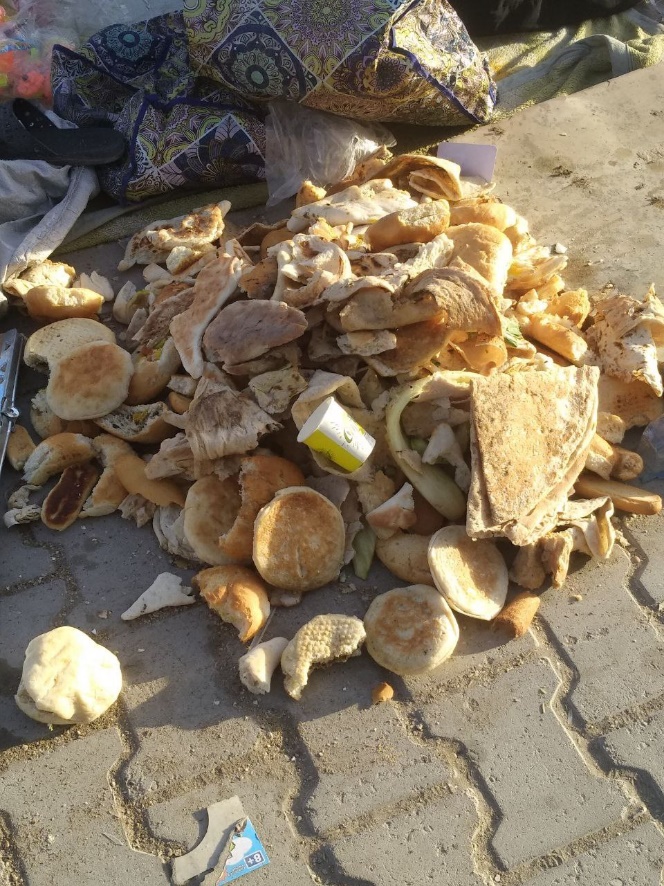 |
| 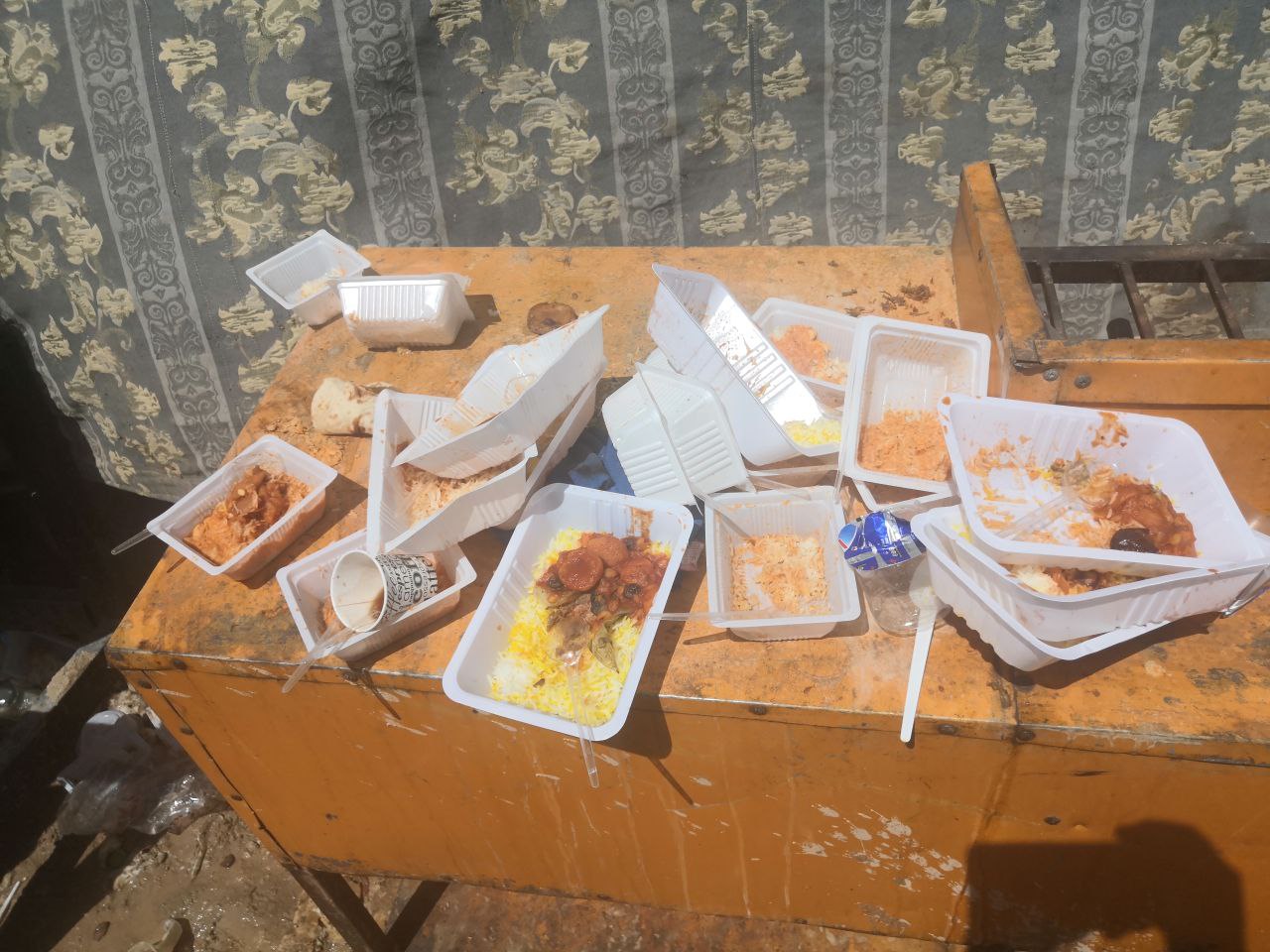 | 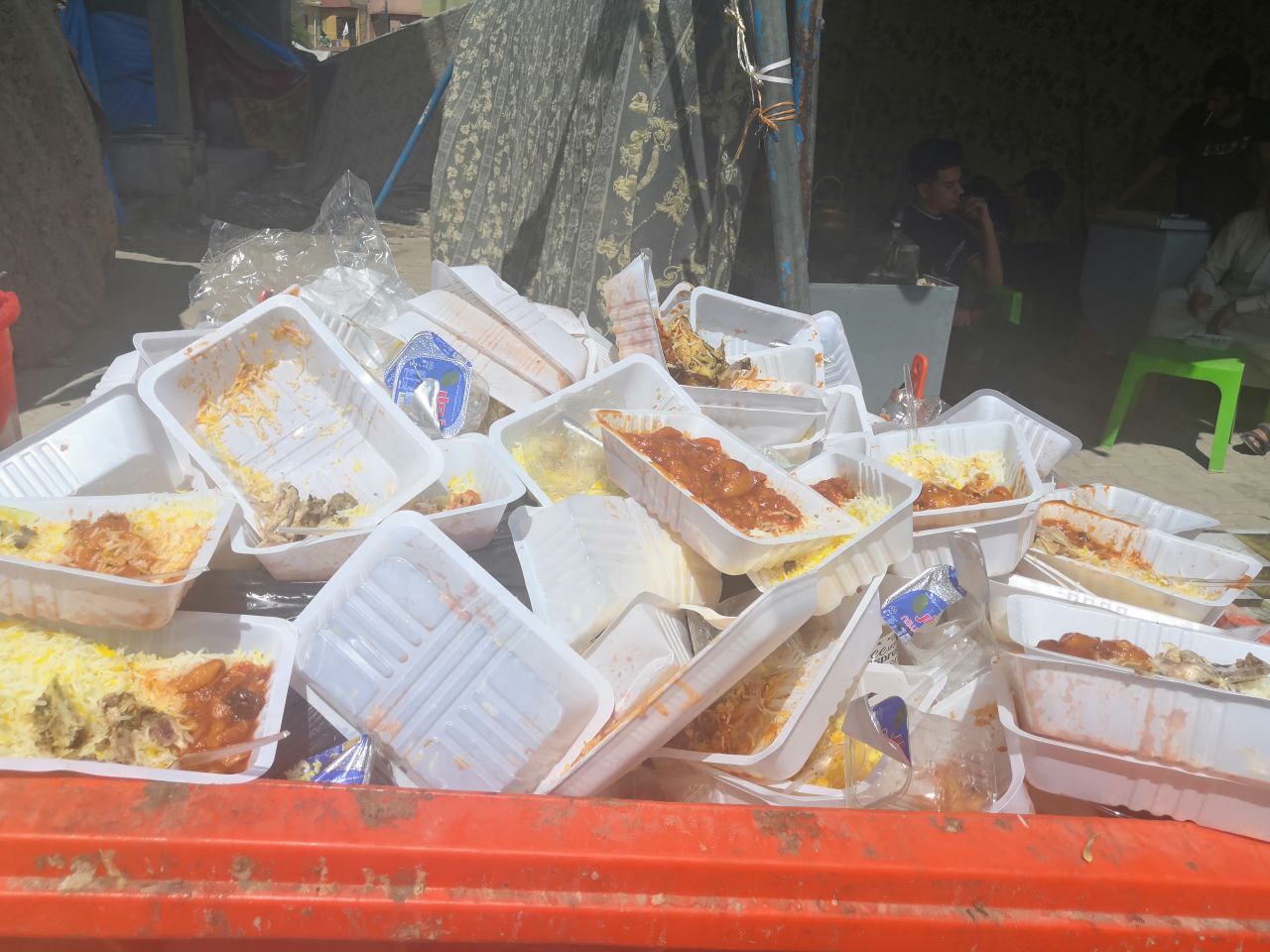 |

Appendix 2. Interview guides, main questions.

MAWKIB OWNERS / OPERATORS

- Tell me about how much food is wasted on your stall, per day or during the festival’s duration. What is the most wasteful day or time of the day and why?
- Tell me what foodstuffs are wasted the most and the reasons behind
- Building on the above, tell me about the main reasons behind food waste on your stall
- What happens to food waste generated in your stall?
- Tell me if you do anything to reduce this food waste? if you do not do anything, what can YOU meaningfully to reduce food waste?
- Tell me how you feel about the food wasted during the festival. What can be done to make the future festivals less wasteful?
- Who should take the lead in food waste reduction in the future festivals and how can YOU help, if at all?

PILGRIMS

- How much food, in your opinion, is wasted during the festival? Tell me how you feel about this wastage.
- What do you think are the main drivers of food waste during the festival?
- Tell me what, in your opinion, can be done to reduce food waste during the future festivals? Who do you think should lead on food waste reduction during the future festivals? What can YOU meaningfully do to reduce food waste during the future festivals?

MUNICIPAL AUTHORITIES

- Tell me about food and plastic waste generated during the festival. How much is wasted? What are the main drivers?
- Our study found that XXX amounts of food and plastic waste are produced. What do you think of these figures?
- What happens to food and plastic waste generated during the festival? What methods of disposal are used and why?
- How do you manage wastage? What efforts are applied, if any, to have this wastage minimized? What about recycling / composting?
- What barriers exist for more effective waste management? What can be done to make it more effective?

RELIGIOUS LEADERS

- Tell me if you know how much food and plastic waste is produced during the festival. If you do not know, what would be your best guess and why?
- Our study found that XXX amounts of food and plastic waste are produced. What do you think of these figures?
- What, in your opinion, are the main drivers of food and plastic waste?
- Waste is forbidden in Islam. Why does, in your opinion, waste still occur in mawkibs and among pilgrims?
- What can be done to minimize wastage? How can behaviour be influenced / changed?
